# Medical News and Bulletin

**Published:** 1843-03-18

**Authors:** 


### PENNSYLVANIA MEDICAL COLLEGE.

At the public Commencement of this institution, held March 7th, the degree of Doctor of Medicine was conferred on twety-three graduates. The valedictory address was delivered by Professor Robert M. Bird.

### JEFFERSON MEDICAL COLLEGE.

At the annual Commencement, held at the Musical Fund Hall, on Friday, March 10th, the degree of Doctor of Medicine was conferred on forty-seven gentlemen. Professor Mitchell delivered the valedictory address.

The following is the act proposed in the Legislature of New York, which will probably become a law; if not during the present session, at no very remote period.

SECTION 1. Any person residing within this State, and being a citizen of the United States, of the age of twenty-one years, assuming to practise physic and surgery, or either, may, after the passage of this act, be entitled to receive for such service a fair compensation, and may sue and collect the same in any court having jurisdiction of like claims for other services; provided, said person shall file and deposit in the clerk's office of the county in which such person resides and designs to practise, a certificate of such intention, setting forth the school of medicine to which such person belongs, and the system upon which he designs to practise. But nothing herein shall be construed to exempt any person refusing or neglecting to comply with the provisions of this act, from the full force and penalties of section 25, Title 7, Chapter 14, of the first Part of the second edition of the Revised Statutes.

§ 2. Any person thus assuming to practise physic and surgery, or either, shall be liable for mal-practice in a suit at law, on the prosecution of any person aggrieved, in any court of this State having cognizance thereof, and be subject to the same pains and penalties as are now provided by existing statutes.

§ 3. So much of the Revised Statutes as is inconsistent with the provisions of this act, is hereby repealed.

§ 4. This act shall take effect immediately.

### *Effects of a Solar Eclipse on Animals.*

By M. ARAGO.

In his report on the eclipse of July 8th, M. Arago mentions in support of a popular notion which he had always disbelieved, that a friend of his put five healthy and lively linnets in a cage together, and fed them immediately before the eclipse. At the end of it three of them were found dead. Other indications of the alarm it produced were seen in a dog which had been long kept fasting, and which was eating hungrily when the eclipse commenced, but left his food as soon as the darkness set in. A colony of ants which had been working actively, suddenly ceased their labors at the same moment.—*Brit. and For. Med. Rev., from Gaz. Med.* Agu. 27.

### *Opportunities of studying Medicine at Brussels.* By JOHN PERKINS, M. D.

Brussels is a large, handsome, airy city, with a climate not unlike that of England, excepting that it is superior in dryness, and that fogs are less frequent and less dense in the former. Living is very cheap here, for although the *market* prices are but little below the smaller towns of Great Britain, yet competition, and the affluence of strangers, have an immense influence upon the amounts for which articles can be bought in Brussels. Decent board and lodging may be obtained by students for 3*l.* a month, and 4*l.* will effect the object handsomely and comfortably. The medical school, forming part of the university, has been established since 1836. The students in it are few, the opportunities numerous. There are two civil hospitals and one military, containing together upwards of eight hundred beds. Excellent lectures on chemistry, botany, mineralogy, comparative and human anatomy, physiology, surgery, and the practice of medicine, are daily given during ten months of the year, and there is a liberal supply of subjects. All the consequent advantages of these things are obtainable for the annual charge of 8*l.* 10s., without extras. The chemical professor, M. Koene, is as skilful as he is indefatigable. Gluge is known as a physiologist to all Europe, and Seutin has recently been demonstrating his system of bandaging in the London hospitals. There are many other teachers in the university on whom I could bestow well-merited praise. In a word, in all branches, except practical medicine, much may be learned; and even in that, the do-nothing system on the one hand, and bleeding and gum-water on the other, may be watched in their effects, whilst pathological anatomy and auscultation are pursued with advantage. As to relaxation, since the bow of science cannot always be strung, pleasant walks and rides in summer; in winter, coffee-houses and clubs of a superior description, and an excellent theatre, afford varied and innocent recreation, on the most moderate terms. The pit of the opera, which may be named immediately after that of Paris or London, costs 1s. 4d., and students are admitted on still lower terms.—*Lon. Lan.*, Jan. 7, 1843.

BAGLIVI used to employ a singular metaphor to convey his notions of the *vis medicatrix naturæ.* He likened the animal frame in disease to a man having fallen into a well, to aid whom everything that comes to hand is thrown in; and, after a multitude of useless matters, he often chances to find some one of these serviceable in helping him to get out. Nature acts in a similar manner against diseases, being ready to derive benefit from the first remedy the effects of which tend toward recovery.—*Ibid*, Jan. 17.

MEDICINES IN GERMANY are subjected to duties determined in an annual tariff. The ordonnance of the Grand Duchy of Baden, on this head, for 1842, fixes the duty on an infusion at one kreutzer per ounce (about a farthing); on the filtration of a pint of liquid, 4 kr.; on an emulsion, 4 kr.; on a packet of powders, 1 kr.; on an extract, or a pillular mass, 6 kr.; a simple mixture, 1 kr. We are at a loss to discover the utility of this to any persons but the tax-eaters.—*Ibid.*

